# Psora as Taught by Hahnemann in the *Chronic Diseases*

**Published:** 1898-04

**Authors:** Eleanor F. Martin


### PSORA, AS TAUGHT BY HAHNEMANN IN THE *CHRONIC DISEASES.*

Eleanor F. Martin, M. D.

Of the three chronic miasms which Hahnemann claims to be the foundation of all chronic diseases, psora is the most important of all. With it, as with the other two, sycosis and syphilis, but one moment is needed for infection; but for the development of this infection, so that it becomes a general disease, invading the entire organism, a longer time is required.

After a number of days have elapsed, the disease in the meantime receiving its fullest internal development so that an outlet seems necessary in order to keep the vital economy from too much injury and consequent danger, kind nature comes to its assistance and brings forth local conditions characteristic of the internal disease which act vicariously for the internal malady, palliating it to a certain extent.

These local conditions, or symptoms, appear on that part of the skin where the miasm touched the nearest nerves during infection; and the skin being the least dangerous part of the body, it can readily be seen how the vitality is saved by this process of nature. Without this cutaneous ailment the disease would not be complete; for this external manifestation is the necessary consequence, though alleviator of the internal malady.

Psora, then, is a chronic miasmatic disease—a complete whole—consisting of an internal invasion of the entire organism, with an external cutaneous eruption.

This is true also of the other two chronic miasms. The same process of nature takes place, and we have the internal invasion with external manifestations as well.

The mistake has been made, and is still being made, that these cutaneous affections are simply local, merely adventitious from without, with no internal connection whatever.

In syphilis the chancre which appears after a certain time following infection is often looked upon as purely local, and to prevent absorption into internal parts it is removed by cauterization. The internal malady, thus robbed of its outlet, is now pent up within the organism, working sad havoc with the general constitution until we soon have a breaking out of the venereal disease internally.

In sycosis the fig-warts characteristic of this disease are cauterized for cure regardless of the internal condition, which is progressing to an increased degree.

So itch has been considered merely a disease of the skin, in which the internal portion of the body takes no part. For this reason the eruption is treated locally, much to the later dissatisfaction of the practitioner and to the great discomfort of the patient, for when this eruption has been destroyed the internal psora is placed in the unnatural position of dominating in a one-sided manner, and is thus compelled to develop secondary symptoms, which usually take the form of most severe chronic ailments.

To cure the entire disease most easily, quickly, and surely the original eruption must be present on the skin; for then the picture of the disease is complete, and a suitable antipsoric remedy given at this time will eradicate the disease—both its internal and external manifestations.

In discussing the cure of psora, there is one distinction to make between it and syphilis and sycosis, namely, in the former condition the cutaneous eruption sometimes disappears from causes other than artificial measures used purposely for its destruction. It may disappear suddenly from the effects of cold, warm baths, the infection of other eruptive diseases, etc., but the internal taint still remains.

In syphilis and sycosis neither the chancre nor fig-warts ever pass away unless destroyed on purpose by external measures, or unless the disease is cured internally.

In the diagnosis and treatment of psora, then, it behooves us to readily understand the condition of our patient, and as readily to act in the work of cure before any accidental factor robs us of part of our disease picture.

The homœopathic physician, however, rarely gets a recent case of itch eruption in his private practice; the patient, on account of the intense burning and itching, usually applying either a home remedy or some local application obtained from a druggist, which eliminates the eruption, and, perhaps, also the opportunity for the homœopathic cure of the disease.

Only in prisons, hospitals, and orphan asylums can cases of itch with the original eruption be met with, and it is here that good homœopathic cures should be made.

The excuse of not knowing how and where infection has been derived is not sufficient for the use of external measures of treatment in cutaneous eruptions of any kind, for it should be remembered that the human skin does not evolve of itself any eruption without the coöperation of the entire organism; and hence should be treated by internal medication to affect the entire organism.

In some cases, as a result of a great change in the organism, the itch eruption reappears on the skin, but it is usually of short duration, passing away in a few days, showing that it lacks the complete quality of the first eruption, and it is therefore unreliable in the thorough cure of the psora.

The poor quality of this secondary eruption is due to the fact that the internal psora has already taken on the form of other chronic ailments, and cannot give to the eruption the force that it could at first.

During the appearance of this secondary eruption the symptoms of the secondary chronic disease, caused by the repression of the first eruption, suddenly disappear, but reappear when this eruption passes away.

It is a mistake, then, after the first eruption has been suppressed, to attempt to produce a secondary eruption in the hope of having some external manifestation to guide in the selection of a remedy, for the transitory characteristics of such secondary external condition convince us of its unreliability as an assistant in the work of cure. Hahnemann himself, before he was fully satisfied with this fact, was in the habit of trying to obtain an artificial renewal of the eruption by checking the respiratory function of the skin, but he found how useless and even harmful such method was.

The remedy *par excellence* that has been used for the cure of itch is Sulphur. Even in ancient times it was looked upon as a sort of specific, but was used only externally in the form of ointments or salves. Baths of warm sulphurous mineral waters were also used; but it soon became apparent that while the patients were freed from the eruption, they were not really cured, but, on the contrary, were soon afflicted with other ailments.

The more modern physicians gave it internally in connection with external measures, and then in large doses of ten, twenty, and thirty grains, frequently repeated, which merely acted as a purgative, and, instead of curing the disease, the external eruption was simply advanced. Often the entire malady increased, or, at least, a new trouble added, partly because the vital force expelled the Sulphur through purging stools or vomiting without having put its healing power to use.

While it is true that many patients seem to be relieved of their symptoms for a time by sulphur baths, they are not really rid of the original disease, but are under the dominion of a sulphur disease which may perhaps be more bearable, and they congratulate themselves that they are at least free from their first trouble. After a time, however, other symptoms arise, often worse than the original ones, and in different parts of the body. They then take a second treatment of baths, only to find less alleviation of their suffering, and often an aggravation. Thus we see that the excessive use of Sulphur in chronic diseases takes away from it all value. The only way that Sulphur can be of service in psora is to give it in potentized preparation when the original itch eruption is present. It will then cure the entire disease both externally and internally, and often with a single dose. It will not cure all conditions of psora. It is only good in the beginning.

As a rule every psoric diathesis, whether still latent or already developed into some chronic disease, is rarely cured by any single anti-psoric remedy. Usually a number of remedies, one after the other, according to the symptoms, are necessary for perfect cure. This seems quite plausible, too, when we consider that psora assumes a great variety of symptoms as it infects individual men with varying bodily constitutions resulting from their peculiar occupations and habits of life, their climatic and hygienic surroundings.

With the medicinal treatment of all chronic diseases the *special* treatment is also very important, much depending upon the judgment of the practitioner. As to the mode of living, circumstances must be yielded to, taking care at the same time that everything be removed which would seem to hinder a cure. The usual daily labors of the patient should be continued in as far as the strength will allow, and only labors which would tend to interfere with the health of healthy persons should be prohibited. Persons whose occupations are of a sedentary nature should be instructed to walk more in the open air, without setting aside their work altogether.

People of the higher classes should be urged to walk more than is their custom. Any innocent amusement should also be allowed. Any act or inclination tending to the excitation of sexuality between the sexes should be interdicted.

In scholars suffering from chronic disorders mental occupation should be limited to work from memory. In mental troubles reading should never be allowed.

All classes of chronic patients must be forbidden the use of domestic remedies, as also the use of strong perfumes and the like. Customary baths should be gradually limited to quick ablutions only so far as cleanliness demands.

The diet must be moderated according to the mode of living of the patient. A plain diet should be insisted upon.

Drinks are usually the most difficult to decide upon. Coffee should be prohibited. In persons over thirty years of age, who have been accustomed to it all their lives, it may be gradually discontinued without causing any particular discomfort to the patient. Rye or wheat roasted and prepared like coffee are good substitutes.

Tea should also be prohibited and some harmless warm drink substituted.

All alcoholic drinks should be gradually stopped or prohibited. Patients who have been accustomed from youth to a plentiful use of such drinks cannot give them up at once or entirely without producing a sudden sinking of their strength, and it might even endanger their lives. In such cases the alcoholic beverage may be mixed with water until but a small quantity of it is actually taken.

Among the articles of diet which are apt to be injurious to chronic patients are dishes containing vinegar or citric acid. These not only cause disagreeable sensations and troubles in many patients, but also antagonize or excessively increase the action of some medicines. Acid and sweet fruits should only be allowed in small quantities.

Articles of diet which might tend to act as palliatives should be prohibited—such as stewed prunes for those inclined to constipation. Moderation in even harmless things is all important.

The use of tobacco and snuffs should be prohibited or gradually diminished.

There are often special hindrances to the cure of chronic diseases which ought to be avoided if possible. The most important of these are uninterrupted grief and vexation. With either one of these two most active destroyers of health and happiness present the best medical treatment will be of little use. Where such a condition exists the mind diseased should first be ministered to by the removal of these annoying factors.

Another obstacle to the cure of chronic diseases is the suppressed sexual instinct in marriageable or married persons of either sex from any cause whatever, and which is usually shown by the many hysterical symptoms leading to melancholia and insanity.

Cases coming from allopathic physicians who have used one or many powerful mixtures, besides prescribing frequent mineral baths for the cure of some chronic ailment, are almost invariably at this stage quite incurable on account of the many disorders that have been added to the original diseases. Only in cases where there is enough vital force left can the physician hope to free it from these secondary conditions, and by careful homœopathic treatment for a considerable time urge on a reassertion of its powers. In these cases the chronic medicinal diseases must first be treated by an improved manner of living and well-regulated diet, with, perhaps, but little medicine. By this method the vital force may change those parts of the organism which it compulsorily degenerated, and the patient may then present a condition similar to the original malady which may be met successfully with anti-psoric remedies.

Another hindrance to the cure of advanced chronic diseases is found in the debility and weakness of the sons of rich parents, who, through their destructive passions and excesses, become mental and physical wrecks; whose perversely treated venereal disease, coupled with a psoric taint, make of them but miserable subjects for the best anti-psoric treatment. One more peculiar obstruction to a cure is where, usually among the lower classes, after repeated infections and repressions of the resulting eruptions, severe chronic ailments have developed from the internal state. A cure, however, may be effected in such patients, provided they are not too much debilitated nor too aged, by a judicious use of anti-psoric remedies, though much more time and patience will be required.

Nature seems to assist also in these difficult cases, for it has been found that in cases of itch arising from a new infection, and regardless of the fact that chronic ailments have rapidly developed from one or more previous infections, if the eruption of the new infection is kept unhindered on the skin, a few doses of the appropriate anti-psoric remedy will cure it as easily and quickly as if it were the first and only infection, and will include in this cure the original taint, with all its secondary ailments.

This is true also in syphilis, where, after local destruction of the chancre or bubo, with consequent breaking out of the venereal disease, a new infection takes place, and the chancre being left undisturbed, a single dose of the best mercurial preparation may cure it, together with the original venereal disease, providing that no complication of sycosis or psora is present; for if this is so they must be treated first.

It is not advisable, however, to intentionally cause an artificial infection of itch in order to secure an easier cure, because usually during severe chronic disorders arising from a psoric origin the itch miasm rarely retains its hold, and seems to cling less when caused by artificial inoculation than when originating from an accidental, unintentional infection.

